# Peer Review of “Prevalence and Determinants of Academic Bullying Among Junior Doctors in Sierra Leone: Cross-Sectional Study”

**DOI:** 10.2196/75134

**Published:** 2025-05-22

**Authors:** Peter Bai James

**Affiliations:** 1 Southern Cross University, Lismore, Australia

**Keywords:** academic bullying, junior doctors, Sierra Leone, mental health, professional development

*This is a peer-review report for “Prevalence and Determinants of Academic Bullying Among Junior Doctors in Sierra Leone: Cross-Sectional Study.”*

## Round 1 Review

### Specific Comments

#### Major Comments

##### Introduction

I think the Introduction in this study [1] needs to be contextualized properly. Saying that bullying in the health care profession has not been looked at is largely correct, but the authors need to strengthen their argument by properly discussing the current literature on bullying in the Sierra Leone educational establishment and the limitations of the current literature as it relates to their topic of enquiry.

Please read the following:

- Osborne A, James PB, Bangura C, Tom Williams SM, Kangbai JB, Lebbieie, A. Bullying victimization among in-school adolescents in Sierra Leone: a cross-sectional analysis of the 2017 Sierra Leone Global School-Based Health Survey. *PLOS Glob Public Health*. Dec 22, 2023;3(12):e0002498. [doi: 10.1371/journal.pgph.0002498] [PMID: 38134001]
- Report on findings from school-related gender-based violence action research in schools and communities in Sierra Leone [2].

##### Methods

I wonder why the authors decided not to recruit all junior doctors who met their inclusion criteria, given that the list of junior doctors in the University of Sierra Leone Teaching Hospitals Complex at the time of data collection can be obtained from each of the constituent teaching hospitals. I know for a fact that the population of junior doctors is not so huge (less than 500). In other words, why did the authors just recruit all 160 junior doctors? Such data can be sourced from the Sierra Leone Medical and Dental Association or from the respective teaching hospital.

What informed the design of the questionnaire used? Why did the authors decide not to conduct any form of validation of the questionnaire (ie, externally or internally) to ensure it is appropriate for the context in which it is used?

This study was among junior doctors, but the authors mentioned registrars. A registrar is no longer a junior doctor. I may be wrong, but I strongly suggest that the authors provide a clear definition of what is the definition of junior doctor in Sierra Leone.

##### Discussion

I beg to disagree. A sample was calculated, and a probabilistic sampling method was used in this study, which means that it gives an equal chance for everyone to be chosen. Thus, the sample used is representative of junior doctors in the University of Sierra Leone Teaching Hospitals Complex. There are two ways to explain your finding: either the sample is not representative because the sampling was not probabilistic or the whole population should have been recruited, or the finding is correct (ie, there are no gender differences).

### Minor Comments

The first two sentences of the third paragraph of the Introduction section: This has already been stated in the previous paragraph. This is just a repetition.
